# The gut microbiome modulates associations between adherence to a Mediterranean-style diet, abdominal adiposity, and C-reactive protein in population-level analysis

**DOI:** 10.1016/j.ajcnut.2023.11.001

**Published:** 2023-11-04

**Authors:** Amy Jennings, Tilman Kühn, Nicola P. Bondonno, Sabina Waniek, Corinna Bang, Andre Franke, Jan Kassubek, Hans-Peter Müller, Marcus Both, Katharina S. Weber, Wolfgang Lieb, Aedín Cassidy

**Affiliations:** 1 Institute for Global Food Security, Queen's University Belfast, Northern Ireland; 2 Heidelberg Institute of Global Health (HIGH), Faculty of Medicine and University Hospital, Heidelberg, Germany; 3 Institute for Nutrition Research, School of Medical and Health Sciences, Edith Cowan University, Perth, Australia; 4 The Danish Cancer Society Research Centre, Copenhagen, Denmark; 5 Institute of Epidemiology and Biobank PopGen, University Hospital Schleswig-Holstein, Campus Kiel and Kiel University, Kiel Germany; 6 Institute of Clinical Molecular Biology, Kiel University, Kiel, Germany; 7 Department of Neurology, University of Ulm, Ulm, Germany; 8 Department of Radiology and Neuroradiology, University Hospital of Schleswig-Holstein, Arnold-Heller-Straße 3, 24105, Kiel, Germany

**Keywords:** diet, gut microbiome, adipose tissue, inflammation

## Abstract

**Background:**

Adherence to a Mediterranean-style dietary pattern is likely to have variable effects on body composition, but the impact of gut microbiome on this relationship is unknown.

**Objectives:**

To examine the potential mediating effect of the gut microbiome on the associations between Alternate Mediterranean Diet (aMed) scores, abdominal adiposity, and inflammation in population-level analysis.

**Design:**

In a community-based sample aged 25 to 83 y (*n =* 620; 41% female) from Northern Germany, we assessed the role of the gut microbiome, sequenced from 16S rRNA genes, on the associations between aMed scores, estimated using validated food-frequency questionnaires, magnetic resonance imaging-determined visceral (VAT) and subcutaneous (SAT) adipose tissue and C-reactive protein (CRP).

**Results:**

Higher aMed scores were associated with lower SAT (-0.86 L (95% CI: -1.56, -0.17), *P* = 0.01), VAT (-0.65 L (95% CI: -1.03,-0.27), *P* = 0.01) and CRP concentrations (-0.35 mg/L; β: −20.1% (95% CI: 35.5, -1.09), *P* = 0.04) in the highest versus lowest tertile after multivariate adjustment. Of the taxa significantly associated with aMed scores, higher abundance of *Porphyromonadaceae* mediated 11.6%, 9.3%, and 8.7% of the associations with lower SAT, VAT, and CRP, respectively. Conversely, a lower abundance of *Peptostreptococcaceae* mediated 13.1% and 18.2% of the association with SAT and CRP levels. Of the individual components of the aMed score, moderate alcohol intake was associated with lower VAT (-0.2 (95% CI: -0.4, -0.1), *P* =0.01) with a higher abundance of *Oxalobacteraceae* and lower abundance of *Burkholderiaceae* explaining 8.3% and 9.6% of this association, respectively.

**Conclusion:**

These novel data suggest that abundance of specific taxa in the *Porphyromonadaceae* and *Peptostreptococcaceae* families may contribute to the association between aMed scores, lower abdominal adipose tissue, and inflammation.

## Introduction

Aging is accompanied by changes in body composition that can negatively affect health, including increases in fat mass, predominantly in the abdominal region. Visceral adipose tissue (VAT) seems particularly important to metabolic and cardiovascular disease risk profiles, including increased odds of hypertension, impaired fasting glucose values, diabetes mellitus, and metabolic syndrome [1]. Furthermore, increased abdominal adiposity induces chronic subclinical inflammation, which contributes to the development and progression of age-related chronic diseases and frailty [2].

A Mediterranean-style dietary pattern is characterized by high intake of plant-based foods (fruit, vegetables, nuts, and cereals) and olive oil, moderate intake of fish and alcohol, and low intake of dairy products and red and processed meat. Data from randomized controlled trials and prospective cohort studies show that Mediterranean-style diets may improve several health parameters, including central obesity [3]. There is limited evidence to suggest if specific components of a Mediterranean-style diet or adherence to the dietary pattern as a whole are responsible for the associations [4]. A Mediterranean-style dietary pattern has also been associated with specific functional and taxonomic components of the gut microbiome, and the limited available data to date suggest that associations between a Mediterranean-style dietary pattern and cardiometabolic health, frailty, and cognitive health may depend on microbial composition [5, 6]. As gut microbiome profiles have been shown to differ between obese and lean individuals, [7] associations between a Mediterranean-style diet and adiposity could be dependent on the composition of the gut microbiome. However, to date, few studies have examined whether the gut microbiome modulates the association between diet and age-related body composition parameters and systemic inflammation.

The aim of the current study was, therefore, to examine the potential mediating effect of the gut microbiome on the associations between adherence to a Mediterranean-style dietary pattern, assessed using Alternate Mediterranean Diet (aMed) scores [8], and clinically measured indicators of aging-related body composition, including magnetic resonance imaging (MRI)-determined volumes of VAT and subcutaneous abdominal fat (SAT) and circulating levels of C-reactive protein (CRP). Due to the diverse nutrient and polyphenol profiles of the plant-based foods included in a Mediterranean-style dietary pattern that are likely to have differing associations with adipose tissue and the gut microbiome, we also examined associations with each individual component of the aMed score.

## Subjects and methods

### Study sample

Participants were part of the PopGen biobank cohort established for research into the genetic risk factors for complex diseases in Kiel, Northern Germany, between 2005 and 2007. The participants in the current analyses compromised the healthy controls recruited from the general population alongside specific patient groups for a range of diseases with likely genotype profiles [9]. The sample of 1316 participants included *n =* 747 from population registries [10] and *n =* 569 blood donors from the University Hospital Schleswig-Holstein in Kiel. The sample is predominately of European descent, but the exact number of individuals belonging to racial and ethnic groups is not known. Regular follow-up examinations with clinical and molecular phenotyping were conducted. The first (2010 to 2012) and second follow-up (2016**–**2017) examinations were attended by 929 and 665 participants, respectively, and comprised biochemical, phenotypic, and dietary assessments.

At the first follow-up assessment, 656 participants agreed to participate in a whole-body MRI examination. Complete data on the MRI variables was available for 626 participants, of which we excluded those who were missing dietary (*n =* 1) or microbiome (*n =* 5) data (Supplemental Figure 1). At the second follow-up assessment, after excluding those with missing dietary or covariate data (*n =* 120), there were complete data for 455 participants on CRP. All participants were unaware of the specific hypotheses being tested and were not selected for particular diseases or traits. The study was approved by the Christian-Albrechts University of Kiel Ethical review board, and all subjects provided informed written consent.

### Adipose tissue assessment

Whole-body MRI was performed on a Magnetom Avanto 1.5-Tesla scanner (Siemens Medical Solutions) with participants in a supine position with arms extended above the head and required to hold their breath to minimize breathing motion artifacts. Transversal images were obtained by a T1-weighted gradient-echo sequence (repetition time 157 ms, time to echo 4 ms, flip angle 70°, voxel size 3.9 × 2.0 × 8.0 mm^3^). Continuous cross-sectional images with 8-mm slice thickness and 2-mm interslice gaps were obtained in the thoracic and abdominal regions.

Data preprocessing and analysis were conducted by the semiautomatic software package ATLAS (Automatic Tissue Labeling Analysis Software, University of Ulm, Germany) [11]. Segmentation of VAT and SAT voxels was performed by using the Adapted Rendering for Tissue Intensity Segmentation algorithm as previously described [11, 12]; both VAT and SAT voxels were measured from the top of the liver to the femoral heads. The number of voxels was multiplied by the voxel size to obtain the volume (L) of VAT and SAT. During postprocessing, liver fat and fat in the intestinal loops and minor MRI artifacts caused mainly by hip implants and stents were excluded from segmented VAT. If MRI artifacts were present that could not be corrected, such as extensive breathing motion, data were excluded from further analysis (*n =* 30).

### CRP

Fasted serum blood samples were obtained from participants in a sitting position. In fresh blood samples, concentrations of CRP were analyzed by enzymatic colorimetry (Roche Diagnostic, Mannheim, Germany) with less than 1 h between blood draw and processing. Values below the limit of detection (0.9 mg/L) were set at the detection limit divided by the square root of 2. Laboratory blood analyses were performed in the laboratory for clinical chemistry of the University Hospital Schleswig-Holstein, Campus Kiel, in Germany.

### Dietary assessment

Dietary intake over the previous year was calculated using a self-administered 112-item food-frequency questionnaire, originally designed and validated for use in the German EPIC study [13]. Intakes of energy and other nutrients were determined using values from the German Food Code and Nutrient Database (version II.3) [14]. We calculated adherence to the aMed score, as developed by Fung et al. [8]. The score is based on the dietary intake of 9 components (vegetables, legumes, fruit, nuts, whole grains, red and processed meat, fish, alcohol, and the ratio of monounsaturated fat to saturated fat). Each component receives one point if intake is above the sex-specific median except for meat (where one point is scored if consumption is less than the median intake). For alcohol, one point is given for intake between 5 to 25 g/d. The final scores range between 0 and 9, with a higher score implying greater adherence. We excluded *n =* 8 participants with implausible energy intakes (<800 kcal/d or >6000 kcal/d).

### Gut microbiome composition analysis

Fecal bacterial DNA was extracted using the QIAamp DNA Stool Mini kit from Qiagen on a QIAcube system. After extraction, the V1-V2 region of the 16S ribosomal RNA gene was sequenced on the MiSeq platform, using the 27F–338R primer pair and dual MID indexing (8 nt each on the forward and reverse primers) with the MiSeq Reagent Kit v3 as previously described [15]. The V1-V2 region has been shown to better assign taxa at the species level compared with other regions [16]. After sequencing, MiSeq fastq files were derived from base calls for read 1 and 2 (R1 and R2), as well as both indices (I1 and I2), using the Bcl2fastq module in CASAVA 1.8.2 with no mismatches in either index sequence allowed. Forward and reverse reads were merged with FLASH software (v1.2) [17], and high-quality data were derived (sequences with <5% nucleotides with quality score >30 performed with fastx toolkit). After removing chimeras in sequences using UCHIME (v6.0), 10,000 reads for each sample were randomly selected [18]. Sequences were clustered at each taxonomical level using the RDP classifier with the latest reference database (RDP14) [19].

### Covariate assessment

Self-reported data on participant characteristics, including sex, age, menopausal status, smoking status, family history of disease, use of medications and dietary supplements, and physical activity, were collected by questionnaire. For physical activity, participants were asked to report the time spent walking, cycling, engaging in sports, and gardening (average of summer and winter seasons), household work, and do-it-yourself activities per week over the past year and the number of flights of stairs climbed per day. The duration of each physical activity was multiplied by the corresponding metabolic equivalent (MET) intensity level and summed for all activities [20]. Weight and height were measured with subjects wearing light clothing and no shoes; 2 kilograms were subtracted to account for clothing.

### Statistical analysis

Participants were ranked into sex-specific tertiles of aMed score and associations with the 9 individual components of the aMed score (vegetables, legumes, fruit, nuts, whole grains, red and processed meat, fish, alcohol, and the ratio of monounsaturated fat to saturated fat) were assessed. Secondly, we determined the associations between sex-specific tertile of aMed score and MRI-determined volumes of SAT, VAT, and CRP as outcome variables (separate model for each outcome) using analysis of covariance (ANCOVA). We also examined the associations between the 9 individual components of the aMed score (vegetables, legumes, fruit, nuts, whole grains, red and processed meat, fish, alcohol, and the ratio of monounsaturated fat to saturated fat) with the above-mentioned outcome variables by comparing mean SAT, VAT, and CRP values in participants above vs. below the sex-specific median intakes of each of the 9 MED score components using ANCOVA. We then examined the association between the aMed score and its 9 components with both taxa abundances and the Shannon index of microbial diversity using ANCOVA. To reduce random error in low abundance taxa, we focused our analysis on the core measurable microbiota, which in this dataset excluded taxa if relative abundance was below 0.01% in at least 10% of samples, leaving 36 taxa at the family level. If we observed statistically significant associations of aMed score or of 1 of its components with diversity measures or taxa, we then assessed the relationship between these microbial factors and SAT, VAT, and CRP using ANCOVA.

All models were adjusted for sex (male, premenopausal females, postmenopausal females), age (y), smoking status (never, former, current), physical activity (METs per week), use of vitamin supplements (y/n), use of hormone therapy (y/n), use of corticosteroids (y/n) and daily intakes of energy (kcal); the adipose tissue parameters were additionally adjusted for height (m) and CRP was additionally adjusted for body mass index (BMI) (kg/m^2^). Due to its skewed distribution, CRP values were natural log-transformed and were presented as geometric means (95% CI) or, where the difference between extreme tertiles are reported, as percentage change in the dependent variable per unit change in the independent variable [100∗ (exp(β) -1)].

We used structural equation modeling to quantify the amount of variation in the association between aMed score (and its components as exposure variables) and SAT, VAT, and CRP values **(**outcome measures**)** that was explained by the microbiome. We considered relative abundance of any taxa significantly associated with both diet and outcome measures (SAT, VAT, and CRP) in the models. We also combined these variables using principal component analysis in order to assess the combined function of the microbiome, considering the first component defined. We presented the results as a ratio of the indirect association—the association between aMed score (exposure) and outcome measures (SAT, VAT, and CRP) mediated by the microbiome, to the total association—the association between aMed score and the microbiome (exposures) on the outcome variables. This represented the proportion of the variance explained by the mediating variable.

Two-sided *P* values < 0.05 were considered statistically significant for all analyses with exception of the microbial taxa abundances, where a multiple testing correction was applied using the Benjamini-Hochberg method for false discovery rate where a *Q*-value <0.25 was considered statistically significant. Statistical analyses were performed with Stata statistical software version 15 (StataCorp, Texas, USA).

## Results

The demographic characteristics and dietary intakes of the 620 participants, aged 25 to 83 y, are shown in Table 1. Mean BMI was 27.2 kg/m^2^, with 28.9 % of participants categorized as overweight or obese with a BMI > 25 kg/m^2^. Of the participants, 37 % of males and 40 % of females were classified as low adherers (0 to 3 points), and 24 % of males and 27 % of females as high adherers (> 6 points) to the aMed score. Higher intakes of all individual components of the aMed score were found across tertile of the aMed score (all *P* < 0.001), except for the red meat and alcohol components (Supplemental Table 1). The greatest associations were observed for the fruit and fish components with standardized linear trends across tertile of the score of 0.53 (95% CI 0.44, 0.62) and 0.62 (95% CI 0.53, 0.70) standard deviations, respectively.

**TABLE 1:** General characteristics, dietary intakes, body fat distribution, and systemic inflammation in our sample (620 males and females from the PopGen cohort)

Characteristic	Adipose tissue data	CRP data
*n*	620	455
Sex, male	368 (59.4%)	164 (54.3%)
Sex, female postmenopausal	181 (29.2%)	160 (35.2%)
Age, y	61.2 (11.8)	64.9 (11.4)
Physical activity, MET/week	105 (64.3)	97.7 (58.6)
Current smoking, yes	72 (11.6%)	34 (11.3%)
BMI, kg/m^2^	27.2 (4.4)	27.7 (4.3)
Height, cm	172 (10.1)	172 (10.0)
SAT, L	6.9 (3.5)	-
VAT, L	4.1 (2.2)	-
CRP, mg/L	-	2.6 (3.8)
Supplement use, yes	246 (39.7%)	106 (35.1%)
Hormone therapy use, yes	22 (3.5%)	7 (2.3%)
Corticosteroids, yes	12 (1.9%)	6 (2.0%)
aMed score, points	4.2 (1.9)	4.4 (1.8)
Vegetables, g/d	201 (93.2)	127 (65.0)
Legumes, g/d	3.2 (3.4)	4.7 (4.0)
Fruit, g/d	229 (150)	172 (132)
Fish, g/d	30.1 (22.9)	21.9 (16.8)
Nuts, g/d	4.4 (5.9)	3.8 (4.9)
Wholegrains, g/d	22.7 (20.1)	28.2 (29.1)
Red meat, g/d	107 (68.6)	120 (98.1)
M: S fat ratio	0.89 (0.11)	0.93 (0.14)
Alcohol, g/d	15.8 (19.6)	16.9 (21.3)
Energy, kcal/d	2295 (679)	2333 (796)

Values are mean (SD). aMed, Alternate Mediterranean Diet; CRP, C-reactive protein; MET, metabolic equivalents; M:S, monounsaturated to saturated; SAT, subcutaneous abdominal adipose tissue; VAT, visceral abdominal adipose tissue;

### Association of aMed score and individual components with indicators of body fat distribution and systemic inflammation.

A higher aMed score was associated with lower SAT, VAT, and CRP (Table 2). After multivariable adjustments, participants in the highest, compared with the lowest, tertile of the aMed score had a 0.86 L lower SAT (95% CI: -1.56, -0.17), a 0.65 L lower VAT (95% CI: -1.03,- 0.27) and 0.35 mg/L lower CRP (β: −20.1%; 95% CI: 35.5, -1.09).

**TABLE 2:** Indicators of body fat distribution and systemic inflammation by sex-specific tertile of Alternate Mediterranean Diet score in our sample (620 males and females from the PopGen cohort)

Outcome variables	T1	T2	T3	*P* value
Subcutaneous abdominal adipose tissue, L	7.48 (7.06,7.91)	6.53 (6.10,6.95)	6.62 (6.09,7.15)	0.01
Visceral abdominal adipose tissue, L	4.46 (4.23,4.70)	3.94 (3.71,4.18)	3.82 (3.52,4.11)	<0.01
CRP, mg/L	1.73 (1.49,1.96)	1.52 (1.27,1.77)	1.38 (1.16,1.60)	0.04

Values are mean (95% CI). All Models were adjusted for sex (male, premenopausal females, postmenopausal females), age (years), smoking status (never, former, current), physical activity (Metabolic equivalents per week), use of vitamin supplements (y/n), use of hormone therapy (y/n), use of corticosteroids (y/n) and daily intakes of energy (kcal); the adipose tissue parameters were additionally adjusted for height (m) and CRP for BMI (kg/m^2^). *P* value, *P*-trend calculated using ANCOVA; CRP, C-reactive protein; T, tertile of Alternate Mediterranean Diet score; Adipose tissue parameters (*n =* 620), *n =* per tertile T1=237, T2=229, T3=154; CRP (*n =* 455), *n =* per tertile T1=192, T2=125, T3=138.

**FIGURE 1:**
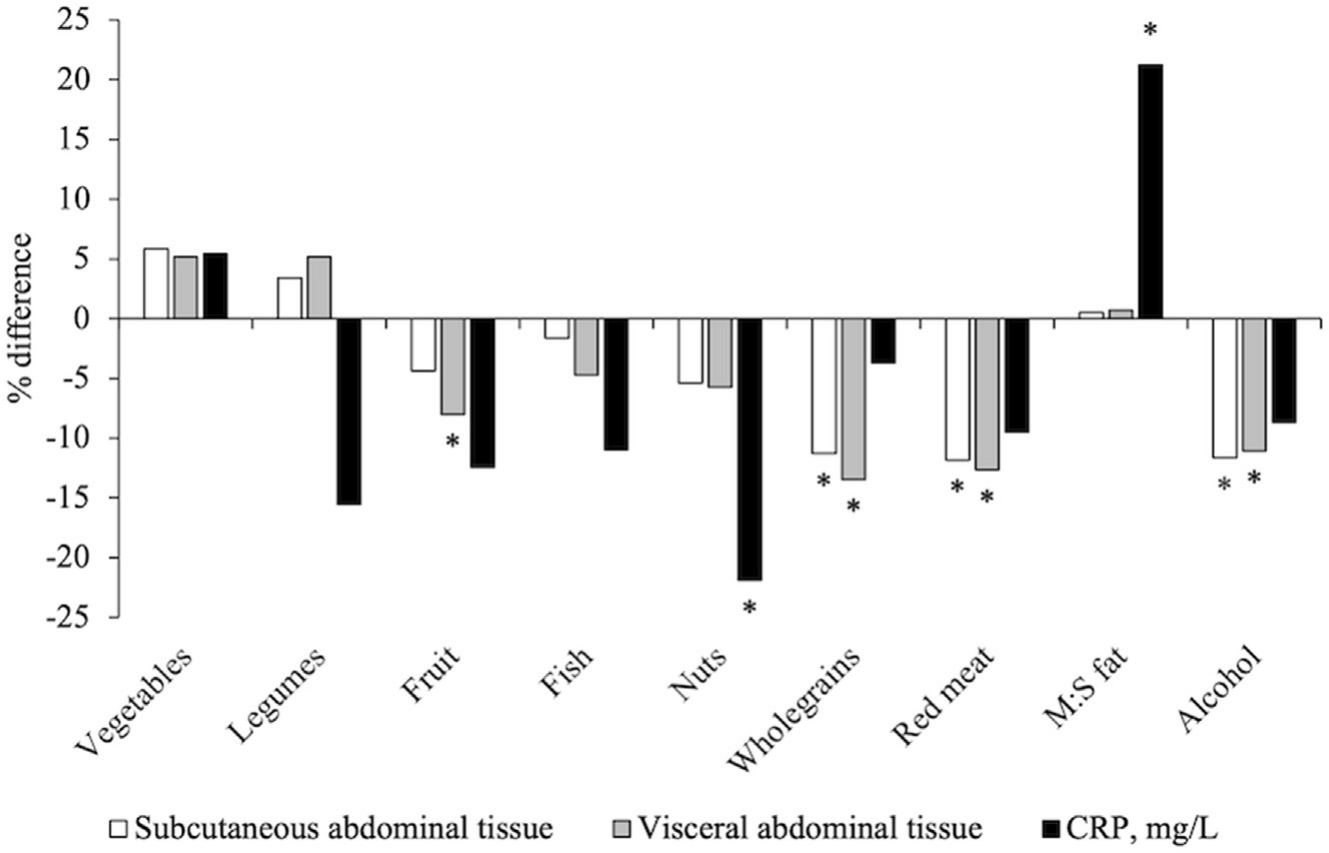
Percentage difference in indicators of body fat distribution and systemic inflammation, comparing participants with intakes above vs. below the sex-specific median for components of the Alternate Mediterranean Diet score in our sample (620 males and females from the PopGen cohort) Bars represent the percentage difference in outcome measures (SAT, VAT, and CRP) between participants with intakes above vs. below the sex-specific median for each component (except the red meat component, which compares participants with intakes below vs. above the median). All models adjusted for sex (male, premenopausal females, postmenopausal females), age (y), smoking status (never, former, current), physical activity (Metabolic equivalents per week), use of vitamin supplements (y/n), use of hormone therapy (y/n), use of corticosteroids (y/n) and daily intakes of energy (kcal); the adipose tissue parameters were additionally adjusted for height (m) and CRP for BMI (kg/m^2^). ∗*P* < 0.05 comparing participants with intakes above vs. below the median for each component, calculated using ANCOVA. Adipose tissue parameters (*n =* 620); CRP (*n =* 455). CRP, C-reactive protein; M:S, monounsaturated fat to saturated fat.

Of the 9 individual components of the aMed score, the strongest associations were observed for lower intake (below the median) of red meat and lower SAT [-0.87 L (95% CI: -1.43, -0.31), *P* < 0.01) and VAT [-0.56 L (95% CI: -0.86, -0.25), *P* < 0.01], moderate alcohol intake and lower SAT [-0.86 L (95% CI: -1.38, -0.33), *P* < 0.01] and higher intake (above the median) of wholegrains and lower VAT [-0.59 L (95% CI: -0.89, -0.30), *P* < 0.01]. Intake above the median of nuts was associated with lower CRP levels [-0.39 mg/L (β: −21.9%; 95% CI: -34.7, -6.7), *P* = 0.01] and a ratio above the median of monounsaturated to saturated fat with higher CRP levels [0.30 mg/L (β: −21.9%; 95% CI: 1.8, 44.3), *P*=0.03] (Figure 1 and Supplemental Table 2).

### Association of aMed score and individual components with gut microbiome composition

A higher aMed score was associated with higher relative abundance of *Porphyromonadaceae* [1.19 (95% CI: 0.17, 2.20), *P* = 0.02 q = 0.24] and lower relative abundance of *Peptostreptococcaceae* [-0.42 (95% CI: -0.71, -0.14), *P* < 0.01 q = 0.13] and *Lactobacillaceae* [-0.10 (95% CI: -0.18, -0.02), *P* = 0.01 q = 0.21], (Table 3). There were no associations between aMed score and alpha diversity, measured using the Shannon index (Table 3), or beta diversity, measured using the Bray–Curtis measure of microbial dissimilarity.

**TABLE 3:** Microbial diversity and relative abundance of taxa by sex-specific tertile of Alternate Mediterranean Diet score in our sample (620 males and females from the PopGen cohort)

Taxa	T1	T2	T3	P	Q
Shannon diversity index	2.62 (2.58,2.67)	2.64 (2.59,2.68)	2.63 (2.58,2.69)	0.78	-
Bray–Curtis (PCoA-1)	0.03 (-0.05,0.11)	0.02 (-0.06,0.10)	0.03 (-0.07,0.13)	0.99	-
Bray–Curtis (PCoA-2)	0.00 (-0.08,0.08)	-0.04 (-0.12,0.04)	-0.06 (-0.16,0.04)	0.40	-
Bray–Curtis (PCoA-3)	0.01 (-0.07,0.09)	0.00 (-0.08,0.08)	0.10 (0.00,0.19)	0.22	-
Bacteroidaceae	17.9 (16.5,19.4)	17.9 (16.4,19.3)	18.1 (16.3,19.9)	0.91	0.98
Rikenellaceae	4.14 (3.63,4.65)	4.47 (3.96,4.99)	4.10 (3.46,4.74)	0.95	0.98
Veillonellaceae	6.14 (5.16,7.12)	5.91 (4.93,6.90)	5.51 (4.28,6.73)	0.44	0.84
Ruminococcaceae	26.7 (25.2,28.1)	26.3 (24.9,27.8)	27.4 (25.6,29.3)	0.58	0.91
Enterobacteriaceae	4.70 (3.40,6.00)	5.05 (3.74,6.36)	4.16 (2.52,5.79)	0.69	0.98
Porphyromonadaceae	4.38 (3.76,5.00)	5.00 (4.37,5.63)	5.57 (4.79,6.35)	0.02	0.24
Lachnospiraceae	14.8 (13.9,15.8)	15.0 (14.0,16.0)	16.2 (15.0,17.4)	0.11	0.49
Verrucomicrobiaceae	0.76 (0.52,1.00)	0.75 (0.51,0.99)	0.75 (0.45,1.05)	0.94	0.98
Sutterellaceae	1.77 (1.41,2.12)	1.49 (1.14,1.85)	1.85 (1.41,2.29)	0.93	0.98
Erysipelotrichaceae	2.88 (2.51,3.25)	2.87 (2.50,3.25)	2.66 (2.19,3.13)	0.52	0.89
Acidaminococcaceae	1.86 (1.48,2.24)	1.43 (1.04,1.81)	1.27 (0.79,1.74)	0.05	0.25
Prevotellaceae	8.19 (6.61,9.77)	8.34 (6.75,9.92)	7.31 (5.33,9.28)	0.55	0.89
Coriobacteriaceae	1.52 (1.26,1.77)	1.55 (1.29,1.81)	1.45 (1.13,1.77)	0.80	0.98
Streptococcaceae	0.52 (0.37,0.68)	0.53 (0.38,0.69)	0.54 (0.34,0.73)	0.91	0.98
Pasteurellaceae	0.52 (0.23,0.81)	0.46 (0.17,0.76)	0.75 (0.39,1.12)	0.40	0.84
Rhodospirillaceae	0.09 (0.05,0.14)	0.06 (0.02,0.11)	0.05 (-0.01,0.11)	0.25	0.65
Desulfovibrionaceae	0.88 (0.72,1.04)	0.69 (0.53,0.85)	0.63 (0.42,0.83)	0.04	0.25
Peptostreptococcaceae	0.80 (0.63,0.98)	0.59 (0.42,0.77)	0.38 (0.16,0.60)	<0.001	0.13
Clostridiaceae	0.47 (0.22,0.72)	0.57 (0.32,0.82)	0.35 (0.04,0.66)	0.65	0.98
Oxalobacteraceae	0.08 (0.03,0.12)	0.10 (0.06,0.14)	0.07 (0.02,0.13)	0.94	0.98
Synergistaceae	0.01 (0.00,0.02)	0.01 (0.01,0.02)	0.01 (0.00,0.02)	0.99	0.99
Clostridiales Incertae Sedis	0.06 (0.05,0.08)	0.05 (0.04,0.07)	0.05 (0.03,0.06)	0.17	0.62
Clostridiales Incertae Sedis XIII	0.19 (0.16,0.23)	0.22 (0.18,0.26)	0.20 (0.15,0.25)	0.74	0.98
Streptophyta	0.02 (0.00,0.04)	0.04 (0.02,0.06)	0.04 (0.01,0.06)	0.32	0.74
Lactobacillaceae	0.13 (0.08,0.18)	0.09 (0.04,0.14)	0.03 (-0.03,0.09)	0.01	0.21
Bifidobacteriaceae	0.07 (0.05,0.10)	0.08 (0.06,0.10)	0.05 (0.03,0.08)	0.33	0.74
Burkholderiaceae	0.02 (0.01,0.02)	0.01 (0.01,0.02)	0.01 (0.01,0.02)	0.23	0.65
Halomonadaceae	0.03 (0.02,0.04)	0.02 (0.01,0.03)	0.01 (0.00,0.02)	0.04	0.25
Shewanellaceae	0.01 (0.00,0.01)	0.00 (0.00,0.01)	0.00 (0.00,0.01)	0.04	0.25
Catabacteriaceae	0.06 (0.04,0.09)	0.07 (0.05,0.10)	0.07 (0.04,0.11)	0.54	0.89
Victivallaceae	0.01 (0.00,0.02)	0.00 (-0.01,0.01)	0.02 (0.01,0.03)	0.13	0.52
Propionibacteriaceae	0.00 (0.00,0.01)	0.00 (0.00,0.01)	0.00 (0.00,0.01)	0.72	0.98
Micrococcaceae	0.00 (0.00,0.01)	0.00 (0.00,0.01)	0.00 (0.00,0.01)	0.22	0.65
Peptococcaceae	0.01 (0.01,0.02)	0.02 (0.01,0.02)	0.01 (0.00,0.02)	0.89	0.98
Christensenellaceae	0.01 (0.00,0.01)	0.01 (0.00,0.01)	0.01 (0.00,0.01)	0.42	0.84
Actinomycetaceae	0.01 (0.01,0.02)	0.01 (0.01,0.02)	0.01 (0.00,0.02)	0.25	0.65

Values are mean (95% CI). Models adjusted for sex (male, premenopausal females, postmenopausal females), age (y), smoking status (never, former, current), physical activity (Metabolic equivalents per wk), use of vitamin supplements (y/n), use of hormone therapy (y/n), use of corticosteroids (y/n) and daily intakes of energy (kcal). *P*, *P*-trend calculated using ANCOVA, Q, false discovery rate adjusted *P* value; PCaA, Principal Coordiante Analysis, T, tertile of Alternate Mediterranean Diet score. *n =* per tertile T1=237, T2=229, T3=154.

Analysis of the relative abundance of genus-level taxa constituting the families associated with aMed scores revealed higher aMed scores were associated with higher relative abundance of *Barnesiella* [0.71 (95% CI: 0.24, 1.19), *P* < 0.01, Supplemental Table 3] of the *Porphyromonadaceae* family and lower relative abundance of *Lactobacillus* [-0.13 (95% CI: -0.22,-0.03), *P* = 0.01].

**FIGURE 2:**
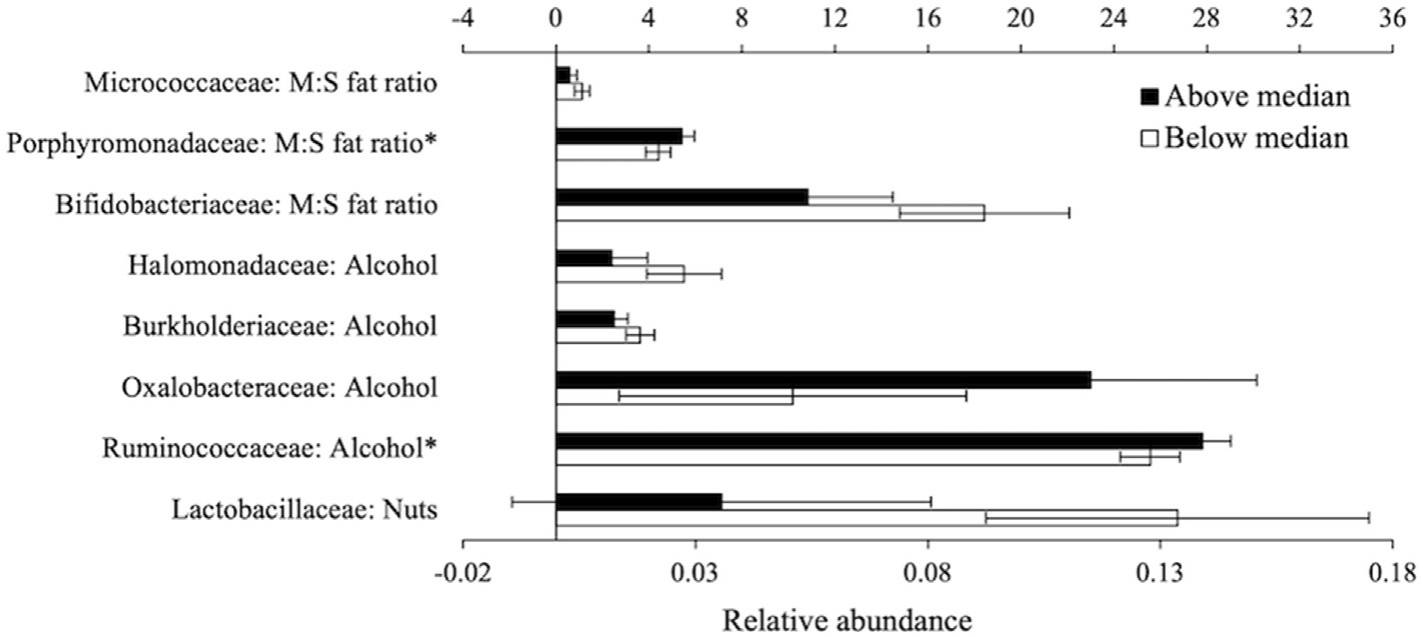
Relative abundance of taxa stratified by sex-specific median for components of the Alternate Mediterranean Diet score in our sample (620 males and females from the PopGen cohort) Bars represent the mean relative abundance, showing only FDR-adjusted significant associations. ∗Bars plotted on the secondary axis. All models adjusted for sex (male, premenopausal females, postmenopausal females), age (y), smoking status (never, former, current), physical activity (Metabolic equivalents per week), use of vitamin supplements (y/n), use of hormone therapy (y/n), use of corticosteroids (y/n) and daily intakes of energy (kcal). M:S, monounsaturated fat to saturated fat.

Of the individual components, a monounsaturated fat to saturated fat ratio above the sex-specific cohort median was associated with a 41% lower relative abundance of *Bifidobacteriaceae*, and 23% and 48% higher relative abundance of *Porphyromonadaceae* and *Micrococcaceae*, respectively, compared to participants below the median. A moderate alcohol intake was associated with 9% higher *Ruminococcaceae*, 126% higher *Oxalobacteraceae*, 31% lower *Burkholderiaceae,* and 56% lower *Halomonadaceae* relative abundance, compared with participants with high or low alcohol intakes. Intake of nuts above the cohort median was associated with a 73% lower relative abundance of Lactobacillaceae (Figure 2).

### Interrelation of aMed score and components, indicators of body fat distribution and systemic inflammation and gut microbiome composition

Of the taxa significantly associated with aMed scores, a higher relative abundance of *Peptostreptococcaceae* and a lower relative abundance of *Porphyromonadaceae* with associated lower SAT, VAT, and CRP levels (Table 4 and Supplemental Figures 2-4). Higher relative abundance of the genus-level taxa *Barnesiella* was associated with lower VAT; likewise, higher relative abundance of *Oxalobacteraceae* and *Burkholderiaceae*, associated with a moderate alcohol intake, were associated with lower and higher VAT, respectively (Table 4).

**TABLE 4:** Associations between relative abundance of taxa and indicators of body fat distribution and systemic inflammation, with path coefficients for associations between Alternate Mediterranean Diet score and components with outcome measures mediated by microbial factors in our sample (620 males and females from the PopGen cohort)

Dietary component	Outcome	Taxa	Association between outcome and taxa		Path coefficients
			Beta (95% CI)	*P*	
aMed score	SAT, L	Peptostreptococcaceae	0.30 (0.11, 0.50)	<0.01	13.1 %
aMed score	SAT, L	Porphyromonadaceae	-0.10 (-0.15, -0.05)	<0.01	11.6 %
aMed score	SAT, L	Lactobacillaceae	-0.34 (-1.03, 0.35)	0.34	-
aMed score	SAT, L	aMed PCA1	-0.57 (-0.82, -0.32)	<0.01	22.6 %
aMed score	SAT, L	Barnesiella	-0.11 (-0.23, 0.01)	0.07	-
aMed score	SAT, L	Lactobacillus	-0.40 (-0.98, 0.18)	0.18	-
aMed score	VAT, L	Peptostreptococcaceae	0.12 (0.02, 0.23)	0.02	6.7%
aMed score	VAT, L	Porphyromonadaceae	-0.06 (-0.09, -0.03)	<0.01	9.3 %
aMed score	VAT, L	Lactobacillaceae	-0.17 (-0.56, 0.21)	0.38	-
aMed score	VAT, L	aMed PCA1	-0.29 (-0.43, -0.15)	<0.01	15.0 %
aMed score	VAT, L	Barnesiella	-0.07 (-0.13, 0.00)	0.04	5.4%
aMed score	VAT, L	Lactobacillus	-0.16 (-0.49, 0.16)	0.32	-
Alcohol	VAT, L	Oxalobacteraceae	-0.74 (-1.18, -0.29)	<0.01	8.3 %
Alcohol	VAT, L	Burkholderiaceae	9.76 (4.30, 15.2)	<0.01	9.6 %
Alcohol	VAT, L	Halomonadaceae	1.14 (-0.94, 3.22)	0.28	-
Alcohol	VAT, L	alcohol PCA1	0.34 (0.20, 0.48)	<0.01	17.2 %
aMed score	CRP, mg/L	Peptostreptococcaceae	0.09 (0.03, 0.15)	<0.01	18.2 %
aMed score	CRP, mg/L	Porphyromonadaceae	-0.02 (-0.04, 0.00)	0.01	-8.7 %
aMed score	CRP, mg/L	Lactobacillaceae	0.09 (-0.01, 0.20)	0.09	—
aMed score	CRP, mg/L	aMed PCA1	-0.14 (-0.22, -0.06)	<0.01	8.7 %
aMed score	CRP, mg/L	Barnesiella	0.01 (-0.04, 0.06)	0.64	—
aMed score	CRP, mg/L	Lactobacillus	0.08 (-0.01, 0.17)	0.09	—
Nuts	CRP, mg/L	Lactobacillaceae	0.09 (-0.01, 0.20)	0.09	—

Values are beta coefficients (95% CI) for the association between each outcome measure (SAT, VAT, and CRP) and microbial taxa shown. PCA1 is the first principal component from analysis of all the microbial factors associated with Alternate Mediterranean Diet score (56% of variation) and alcohol (52% of variation) and at least one outcome variable. Models adjusted for sex (male, premenopausal females, postmenopausal females), age (y), smoking status (never, former, current), physical activity (Metabolic equivalents per week), use of vitamin supplements (y/n), use of hormone therapy (y/n), use of corticosteroids (y/n) and daily intakes of energy (kcal); the adipose tissue parameters were additionally adjusted for height (m) and CRP for BMI (kg/m^2^). *P* value= *P*-trend across tertile of aMed score or comparing participants with intakes above vs. below the median for each component, calculated using ANCOVA. Path coefficients, calculated using structural equation modeling, are for the ratio of the indirect to total association between Alternate Mediterranean Diet score and components, microbial factors, and outcome measures (SAT, VAT, and CRP). The total association is the association between dietary component and outcome measures **(**SAT, VAT, and CRP**)** controlling for microbial factors, the indirect association is the association between dietary component and outcome measures **(**SAT, VAT, and CRP**)** explained by microbial factors and percentage values (shown) are the ratio of the indirect to total association and represent the percentage variation explained by the microbial factors. Adipose tissue parameters (*n =* 620); CRP (*n =* 455). aMed, Alternate Mediterranean Diet; CRP, C-reactive protein; M:S, monounsaturated fat to saturated fat; PCA1, first principal component; SAT, subcutaneous abdominal adipose tissue; VAT, visceral abdominal adipose tissue

The proportion of the association between aMed scores and VAT, SAT, and CRP, respectively, that could be explained by the gut microbiome was 6.7 %, 13.1 %, and 18.2 % for *Peptostreptococcaceae* and 9.3 %, 11.6 %, and 8.7 % for *Porphyromonadaceae* (Table 4 and Supplemental Figures 2–4). A linear combination of the gut microbiome variables associated with aMed scores [first principal component (aMed PCA1; 56 % of the variance)] explained 15 %, 22.6 %, and 8.7 % of the association with VAT, SAT, and CRP, respectively. For the association between moderate alcohol intake and VAT, *Oxalobacteraceae* and *Burkholderiaceae* explained 8.3 % and 9.6 % of the association, respectively, and the linear combination of gut microbiome variables associated with moderate alcohol intake (first principal component: 52 % of the variance) 17.2 %.

## Discussion

To our knowledge, to date, no study has directly investigated the interrelations between adherence to a Mediterranean-style diet, MRI-determined volumes of VAT and SAT, currently a gold standard technique for the quantitative assessment of intraabdominal adipose tissue [21], and the gut microbiome in a community-based sample.

Our data suggest that up to 23 % of the association between aMed scores and MRI-determined abdominal fat and inflammation could be explained by mediation by the gut microbiome. Specifically, higher aMed scores were associated with 12 % (0.65 L) lower VAT and 20 % (0.22 mg/L) lower CRP, and 23 % and 9 % of these associations, respectively, could be explained by mediation with higher relative abundance of *Porphyromonadaceae* and lower relative abundance of *Peptostreptococcaceae*. Microbiome profiles enriched with *Porphyromonadaceae* have previously been associated with lower levels of VAT in a small cohort of older adults [22]. In another study, increased abundance of Peptostreptococcaceae has been associated with nonalcoholic fatty liver disease [23] and, in rat models, with reduced lifespan [24] and increased fasting and postprandial blood glucose values and liver fat accumulation [25]. These taxa likely play a role as adiposity modulators through the production of short-chain fatty acids, and in particular, a higher abundance of *Porphyromonadaceae* has been associated with higher levels of acetate, n-Butyrate, and propionate [26]. Short-chain fatty acids are thought to preserve intestinal barrier function by inhibiting the passage of proinflammatory molecules into the systemic circulation and preventing the occurrence of metabolic endotoxemia [27, 28]. Butyrate has also been shown to have a direct effect on adipose tissue by promoting uncoupling protein-1 expression and adaptive thermogenesis in mice with a depleted gut microbiome [29]. Microbial taxa belonging to the Peptostreptococcaceae family have been shown to be positively associated with blood levels of trimethylamine *N*-oxide [30]. Given that high circulating levels of trimethylamine *N*-oxide and its dietary precursor, choline, have been associated with low adherence to a Mediterranean-style dietary pattern and higher SAT and VAT [31, 32], this is likely another potential underlying mechanism explaining these associations.

We also found associations between higher aMed scores and lower abundance of *Lactobacillus*, although this taxon did not mediate associations with abdominal fat. In animal studies, higher *Lactobacillus* abundance has been shown in Mediterranean-style diets versus Western diet groups, although this was mediated by body weight, with lean animals having the highest abundance regardless of diet group [33]. The genus *Lactobacillus* has been shown to encompass an unusually diverse number of species, which differ according to characteristics such as age, region, and diet and have differential effects on health endpoints, including body weight [34, 35]. Future studies should consider species-level analysis to understand the associations between diet, *Lactobacillus* abundance, and body weight.

Of the individual components of the aMed score, a moderate alcohol intake was associated with 11 % (0.48 L) lower VAT, and 17 % of this association could be explained by gut microbial factors, including *Oxalobacteraceae* abundance which has previously been reported to be higher in leaner individuals [36]. A higher ratio of monounsaturated to saturated fat was associated with higher CRP levels and lower abundance of the *Bifidobacterium* taxa. The reasons for the association with higher CRP are unclear, although higher intakes of monounsaturated fat have previously been associated with lower abundance of *Bifidobacteriaceae*, which, although not confirmed in this study, have been associated with higher inflammatory markers, including CRP [37, 38].

Associations between adherence to a Mediterranean-style dietary pattern and Prevotella species, particularly *Prevotella copri* have previously been reported in a number of studies [5, 6, 39] but were not confirmed in the current analysis. Although these previous studies reported that adherence to the Mediterranean-style diet modulated *Prevotella copri* with subsequent reductions in cardiometabolic risk and improved cognitive function, it appears that, due to the unusual ecological distribution patterns in Western populations, with the taxa absent in most individuals, that relative abundance of this taxa is unlikely to be linearly associated with health outcomes [5, 40].

A Mediterranean-style diet is characterized by high polyphenolic-rich food intake, including olive oil, nuts, red wine, vegetables, fruits, legumes, and whole-grain cereals. The gut microbiome, polyphenolic intake, and metabolism are interrelated, resulting in modulation of both the gut microbiome and the compound structures, which are catabolized into metabolites with increased bioactivity [41]. As a result of the individual components of the Mediterranean dietary pattern, we would expect whole grains, vegetables, and fruits to have driven these associations, especially as these foods are high in fiber, which is known to modify the composition of the microbiome [42]. It was, therefore, surprising that only the association between moderate alcohol intake and VAT could be explained by the gut microbiome, while the ratio of mono- to un-saturated fat was associated with the gut microbiome profile, these taxa did not mediate the association with outcome variables. We have previously shown in this cohort that higher consumption of foods rich in anthocyanins (including red wine) was associated with lower VAT and that microbial diversity and higher abundance of butyrate-producing taxa explained 18.5 % of this association [12].

Strengths of the current study include our ability to examine associations and inter-relationships between aMed scores, outcome measures (SAT, VAT, and CRP), and the profile of the gut microbiome concurrently. In particular, the direct measurement and quantification of adipose tissue using MRI, including the use of an unbiased postprocessing approach, are a particular strength. In addition, these data were collected from a large and well-characterized community-dwelling sample with an extensive range of important known confounders of variation in the gut microbiome. The following limitations merit consideration: this was a cross-sectional study, so we were unable to infer causation from the findings and cannot determine whether the interactions between diet and the gut microbiome are a cause or a consequence of the development of excess adipose tissue and high CRP levels, highlighting the need for mechanistic studies. Studies have shown obesity to be associated with characteristic changes in the gut microbiome with the transfer of an obese microbiota to lean germ-free animals, resulting in increased fat mass [43]. Furthermore, although the use of self-reported dietary assessment has been questioned [44], it is well established that food-frequency questionnaires are valid in ranking individuals appropriately to examine associations between food intake and health endpoints in large cohorts, in particular for dietary patterns [45]. For our analyses, we ranked participants according to tertile of the aMed score or by the median value of the individual components. In addition, we used one inflammatory biomarker, and although CRP is a strong predictor of abdominal fat, other markers, such as IL-6, are also important [46]. Finally, as with all observational studies, despite our detailed adjustment for a range of dietary and lifestyle variables, there is still the possibility of residual or unmeasured confounding from additional, unmeasured factors.

In conclusion, we have shown, for the first time, that participants with higher aMed scores had lower levels of adipose tissue and systemic inflammation, higher relative abundance of *Porphyromonadaceae,* and lower relative abundance of *Peptostreptococcaceae,* compared with those with the lowest scores. We estimated that up to 20% of the association between Mediterranean-style diet adherence and adiposity and inflammation could be explained by mediation with the gut microbiome. This suggests that future trials should consider the interindividual variability of the gut microbiome on moderating the effect of dietary patterns on health outcomes to further develop precision nutrition approaches.

### Author contributions

The authors’ responsibilities were as follows—AJ, WL, and AC: designed the study; WL, SB and KSW: collected the data; CB and AF: conducted the microbiome assessment; HM, JK and MB: provided the ATLAS and advised in the course of MRI analysis; AJ: performed the statistical analysis; AJ and AC: drafted the paper; TK, NB, HM, JK and WL: provided critical review of the manuscript; and all authors: read and approved the final manuscript. AC has primary responsibility for the final content.

### Conflict of interest

The authors declare that they have no conflict of interest.

### Funding

Supported in part by Deutsche Forschungsgemeinschaft (German Research Foundation) grant EXC 22167-390884018 (to WL) under Germany’s Excellence Strategy and German Federal Ministry of Education and Research grant 01GR0468. The PopGen 2.0 network is supported by the German Federal Ministry of Education and Research grant 01EY1103 (to WL) and the Medical Faculty of the University of Kiel.

### Data availability

The data that support the findings of this study are available from the corresponding author upon reasonable request.
