# Supplementary material for: The gut microbiome modulates associations between adherence to a Mediterranean-style diet, abdominal adiposity, and C-reactive protein in population-level analysis
Source: Am J Clin Nutr. 2023 Nov 4;119(1):136–44. doi: 10.1016/j.ajcnut.2023.11.001 (PMC10808821; doi:10.1016/j.ajcnut.2023.11.001)
Supplement: Multimedia component 1 [file mmc1.docx]

**Supplementary materials**

**Supplemental Figure 1:** **Flow chart of the PopGen study population**

**Supplemental Figure 2:** **Path coefficients for associations between Alternate Mediterranean Diet score and volumes of subcutaneous abdominal adipose tissue mediated by microbial factors in 620 males and females from the PopGen cohort**

**Supplemental Figure 3:** **Path coefficients for associations between Alternate Mediterranean Diet score and volumes of visceral abdominal adipose tissue mediated by microbial factors in 620 males and females from the PopGen cohort**

**Supplemental Figure 4:** **Path coefficients for associations between Alternate Mediterranean Diet score and C-reactive protein mediated by microbial factors in 455 males and females from the PopGen cohort**

**Supplemental Table 1: Mean intakes of Alternate Mediterranean Diet score component by sex-specific tertile of total score in 620 males and females from the PopGen cohort.**

**Supplemental Table 2: Indicators of body fat distribution and systemic inflammation by Alternate Mediterranean Diet score component in 620 males and females from the PopGen cohort.**

**Supplemental Table 3: Relative abundance of genus-level taxa in the *Porphyromonadaceae* and *Peptostreptococcaceae* families by sex-specific tertile of Alternate Mediterranean Diet score in our sample (620 males and females from the PopGen cohort).**

**Supplemental Figure 1:** **Flow chart of the PopGen study population**

PopGen control cohort (recruited 2005 - 2007)

**n= 1316**

First follow-up examination (2010-2012)

**n= 929**

Attended for MRI examination

**n= 656**

Incomplete MRI data or containing artefacts (n= 30)

Missing data:

FFQ (n= 1)

Microbiome (n= 5)

Included in adipose tissue analyses

**n= 620**

Included in CRP analyses:

**n= 455**

Second follow-up examination (2016-2017)

**n= 665**

Missing data:

FFQ (n= 59)

Microbiome (n= 34)

BMI (n=1)

Physical activity (n=18)

CRP (n=90)

Implausible energy intake (n=8)

**Supplemental Figure 2:** **Path coefficients for associations between Alternate Mediterranean Diet score and volumes of subcutaneous abdominal adipose tissue mediated by microbial factors in 620 males and females from the PopGen cohort.**

Values are the path coefficients, calculated using structural equation modelling, for the total and indirect associations. Values in bold represent the total association between

Alternate Mediterranean Diet score and subcutaneous abdominal adipose tissue controlling for microbial factors; other values represent the indirect association between Alternate Mediterranean Diet score and subcutaneous abdominal adipose tissue explained by microbial factors; percentage values are the ratio of the indirect to the total association and represent the percentage of variation explained by the microbial factors. PCA1 is the first principal component from analysis of all the microbial factors associated with Alternate Mediterranean Diet score (56% of variation). Models adjusted for sex (male, pre-menopausal females, post-menopausal females), age (years), smoking status (never, former, current), physical activity (Metabolic equivalents per week), use of vitamin supplements (y/n), use of hormone therapy (y/n), use of corticosteroids (y/n), daily intakes of energy (kcal) and height (m). aMed= Alternate Mediterranean Diet*;* PCA= Principal Component Analysis; SAT= Subcutaneous abdominal adipose tissue.

*Peptostreptococcaceae*

*Porphyromonadaceae*

PCA1

**-0.19**

-0.03 (13.1 %)

-0.02 (11.6 %)

-0.04 (22.6 %)

**Supplemental Figure 3:** **Path coefficients for associations between Alternate Mediterranean Diet score and volumes of visceral abdominal adipose tissue mediated by microbial factors in 620 males and females from the PopGen cohort.**

Values are the path coefficients, calculated using structural equation modelling, for the total and indirect associations. Values in bold represent the total association between

Alternate Mediterranean Diet score and visceral abdominal adipose tissue controlling for microbial factors; other values represent the indirect association between Alternate Mediterranean Diet score and visceral abdominal adipose tissue explained by microbial factors; percentage values are the ratio of the indirect to the total association and represent the percentage of variation explained by the microbial factors. PCA1 is the first principal component from analysis of all the microbial factors associated with Alternate Mediterranean Diet score (56% of variation). Models adjusted for sex (male, pre-menopausal females, post-menopausal females), age (years), smoking status (never, former, current), physical activity (Metabolic equivalents per week), use of vitamin supplements (y/n), use of hormone therapy (y/n), use of corticosteroids (y/n), daily intakes of energy (kcal) and height (m). aMed= Alternate Mediterranean Diet; PCA= Principal Component Analysis; VAT= Visceral abdominal adipose tissue.

*Peptostreptococcaceae*

*Porphyromonadaceae*

PCA1

-0.14

-0.01 (6.7 %)

-0.01 (9.3 %)

-0.02 (15.0 %)

*Barnesiella*

-0.01 (5.4 %)

**Supplemental Figure 4:** **Path coefficients for associations between Alternate Mediterranean Diet score and C-reactive protein mediated by microbial factors in 455 males and females from the PopGen cohort**

Values are the path coefficients, calculated using structural equation modelling, for the total and indirect associations. Values in bold represent the total association between

Alternate Mediterranean Diet score and C-reactive protein controlling for microbial factors; other values represent the indirect association between Alternate Mediterranean Diet score and C-reactive protein explained by microbial factors; percentage values are the ratio of the indirect to the total association and represent the percentage of variation explained by the microbial factors. PCA1 is the first principal component from analysis of all the microbial factors associated with Alternate Mediterranean Diet score (56% of variation). Models adjusted for sex (male, pre-menopausal females, post-menopausal females), age (years), smoking status (never, former, current), physical activity (Metabolic equivalents per week), use of vitamin supplements (y/n), use of hormone therapy (y/n), use of corticosteroids (y/n), daily intakes of energy (kcal) and BMI (kg/m^2^). aMed= Alternate Mediterranean Diet*;* CRP= C-reactive protein; PCA= Principal Component Analysis.

*Peptostreptococcaceae*

*Porphyromonadaceae*

PCA1

-0.04

-0.01 (18.2 %)

-0.004 (8.7 %)

-0.003 (8.7 %)

**Supplemental Table 1: Mean intakes of Alternate Mediterranean Diet score component by sex-specific tertile of total score in 620 males and females from the PopGen cohort.**

| **Component** | **T1 (n=237)** | **T2 (n=229)** | **T3 (n=154)** | **Linear trend** | **P-trend** |
| --- | --- | --- | --- | --- | --- |
|  | Mean (95% CI) | Mean (95% CI) | Mean (95% CI) | Standardized β (95% CI) |  |
| **Vegetables** | **160 (149, 171)** | **207 (196, 218)** | **255 (242, 269)** | **0.51 (0.42, 0.60)** | **<0.001** |
| **Legumes** | **2.14 (1.72, 2.55)** | **3.27 (2.85, 3.69)** | **4.83 (4.31, 5.35)** | **0.39 (0.29, 0.48)** | **<0.001** |
| **Fruit** | **154 (137, 172)** | **252 (234, 270)** | **311 (289, 333)** | **0.53 (0.44, 0.62)** | **<0.001** |
| **Fish** | **19.6 (17.1, 22.1)** | **28.4 (25.8, 31.0)** | **48.6 (45.5, 51.8)** | **0.62 (0.53, 0.70)** | **<0.001** |
| **Nuts** | **2.21 (1.53, 2.90)** | **4.07 (3.37, 4.77)** | **8.32 (7.47, 9.17)** | **0.50 (0.41, 0.60)** | **<0.001** |
| **Wholegrains** | **44.4 (40.1, 48.7)** | **58.3 (53.9, 62.7)** | **57.8 (52.4, 63.2)** | **0.21 (0.11, 0.31)** | **<0.001** |
| Red meat | 111 (102, 119) | 106 (97.2, 115) | 101 (90.6, 112) | -0.07 (-0.17, 0.03) | 0.19 |
| **M:S fat ratio** | **0.87 (0.85, 0.88)** | **0.88 (0.87, 0.90)** | **0.95 (0.94, 0.97)** | **0.36 (0.26, 0.46)** | **<0.001** |
| Alcohol | 16.6 (14.1, 19.1) | 15.8 (13.3, 18.4) | 14.5 (11.4, 17.6) | -0.05 (-0.15, 0.05) | 0.32 |

Values are unadjusted means (95% CI) or standardised beta coefficients (95% CI) and *P*-trend across tertile of score.

M:S= monounsaturated fat to saturated fat

**Supplemental Table 2: Indicators of body fat distribution and systemic inflammation by Alternate Mediterranean Diet score component in 620 males and females from the PopGen cohort.**

| **Measure of frailty** | **Component** | **β (95% CI)** | **P=** |
| --- | --- | --- | --- |
| Subcutaneous abdominal adipose tissue, dm^3^ | Vegetables | 0.39 (-0.15, 0.93) | 0.16 |
|  | Legumes | 0.23 (-0.35, 0.82) | 0.44 |
|  | Fruit | -0.31 (-0.85, 0.23) | 0.26 |
|  | Fish | -0.11 (-0.68, 0.45) | 0.70 |
|  | Nuts | -0.38 (-0.92, 0.16) | 0.16 |
|  | **Wholegrains** | **-0.83 (-1.36, -0.29)** | **<0.01** |
|  | **Red meat** | **-0.87 (-1.43, -0.31)** | **<0.01** |
|  | M:S fat ratio | 0.04 (-0.50, 0.57) | 0.90 |
|  | **Alcohol** | **-0.86 (-1.38, -0.33)** | **<0.01** |
| Visceral abdominal adipose tissue, dm^3^ | Vegetables | 0.21 (-0.09, 0.51) | 0.17 |
|  | Legumes | 0.21 (-0.11, 0.53) | 0.20 |
|  | **Fruit** | **-0.34 (-0.64, -0.04)** | **0.02** |
|  | Fish | -0.20 (-0.51, 0.11) | 0.21 |
|  | Nuts | -0.24 (-0.54, 0.06) | 0.11 |
|  | **Wholegrains** | **-0.59 (-0.89, -0.30)** | **<0.01** |
|  | **Red meat** | **-0.56 (-0.86, -0.25)** | **<0.01** |
|  | M:S fat ratio | 0.03 (-0.27, 0.32) | 0.84 |
|  | **Alcohol** | **-0.48 (-0.77, -0.19)** | **<0.01** |
| CRP, mg/L | Vegetables | 0.05 (-0.13, 0.23) | 0.57 |
|  | Legumes | -0.17 (-0.34, 0.01) | 0.06 |
|  | Fruit | -0.13 (-0.31, 0.04) | 0.14 |
|  | Fish | -0.12 (-0.29, 0.06) | 0.20 |
|  | **Nuts** | **-0.25 (-0.43, -0.07)** | **0.01** |
|  | Wholegrains | -0.04 (-0.22, 0.14) | 0.68 |
|  | Red meat | -0.10 (-0.29, 0.09) | 0.31 |
|  | **M:S fat ratio** | **0.19 (0.02, 0.37)** | **0.03** |
|  | Alcohol | -0.09 (-0.27, 0.09) | 0.31 |

Values are β coefficients (95% CI) comparing participants with intakes above vs. below the median for each component (except the red meat component which compare participants with intakes below vs. above the median). All Models adjusted for sex (male, pre-menopausal females, post-menopausal females), age (years), smoking status (never, former, current), physical activity (METs per week), use of vitamin supplements (y/n), use of hormone replacement therapy (y/n), use of corticosteroids (y/n) and daily intakes of energy (kcal); the adipose tissue parameters were additionally adjusted for height (m) and CRP for BMI (kg/m2). P-value= compares participants with intakes above vs. below the median for each component, calculated using ANCOVA. Adipose tissue parameters (n=620); CRP (n=428). CRP= C-reactive protein; M:S=monounsaturated fat to saturated fat.

**Supplemental Table 3: Relative abundance of genus-level taxa in the *Porphyromonadaceae* and *Peptostreptococcaceae* families by sex-specific tertile of Alternate Mediterranean Diet score in our sample (620 males and females from the PopGen cohort).**

| **Taxa** | **T1** | **T2** | **T3** | **P=** |
| --- | --- | --- | --- | --- |
| ***Barnesiella*** | **1.09 (0.80,1.38)** | **1.36 (1.07,1.66)** | **1.80 (1.44,2.17)** | **<0.01** |
| *Butyricimonas* | 0.14 (0.11,0.18) | 0.15 (0.11,0.18) | 0.13 (0.08,0.17) | 0.58 |
| *Odoribacter* | 0.20 (0.15,0.24) | 0.22 (0.17,0.26) | 0.17 (0.11,0.23) | 0.59 |
| *Parabacteroides* | 2.39 (2.09,2.70) | 2.28 (1.97,2.58) | 2.34 (1.96,2.72) | 0.79 |
| ***Lactobacillus*** | **0.16 (0.10,0.22)** | **0.10 (0.04,0.16)** | **0.03 (-0.04,0.11)** | **0.01** |

Values are mean (95% CI). Models adjusted for sex (male, pre-menopausal females, post-menopausal females), age (years), smoking status (never, former, current), physical activity (METs per week), use of vitamin supplements (y/n), use of hormone replacement therapy (y/n), use of corticosteroids (y/n) and daily intakes of energy (kcal). P = *P*-trend calculated using ANCOVA. T= tertile of Alternate Mediterranean Diet score. n= per tertile T1=237, T2=229, T3=154.
